# Supplementary material for: An Inhalable Theranostic System for Local Tuberculosis Treatment Containing an Isoniazid Loaded Metal Organic Framework Fe-MIL-101-NH2—From Raw MOF to Drug Delivery System
Source: Pharmaceutics. 2019 Dec 17;11(12):687. doi: 10.3390/pharmaceutics11120687 (PMC6969914; doi:10.3390/pharmaceutics11120687)
Supplement: Supplementary file 1 [file pharmaceutics-11-00687-s001.pdf]

pharmaceutics

Supplementary Materials: An Inhalable Theranostic System for Local Tuberculosis Treatment Containing an Isoniazid Loaded Metal Organic Framework Fe-MIL-101-NH2—From Raw MOF to Drug Delivery System

Gabriela Wyszogrodzka-Gaweł, Przemysław Dorożyński, Stefano Giovagnoli, Weronika Strzempek, Edyta Pesta, Władysław P. Węglarz, Barbara Gil, Elżbieta Menaszek and Piotr Kulinowski

Table S1. ANOVA statistics and coefficients for the models obtained by the 2^3 factorial design. See section 5.4 and Table 1 for equation terms and factors definitions.

Response	FPF (%)				ED (%)			
Factors	Coefficient Estimate	Mean Square	F Value	p-value	Coefficient Estimate	Mean Square	F Value	p-value
Intercept	0.14	2.034×10^{-3}	129.87	<0.0001	93.62	4.17	16.96	0.0013
A	−2.766E-003	6.121×10^{-5}	3.91	0.0886	0.69	3.80	15.43	0.0044
B	0.024	4.736×10^{-3}	302.45	<0.0001	-	-	-	-
A^2	0.024	1.304×10^{-3}	83.25	<0.0001	−1.44	4.55	18.49	0.0026
Residual	-	1.566×10^{-5}	-	-	-	0.25	-	-
Lack of Fit	-	1.270×10^{-5}	0.55	0.7445	-	0.29	2.41	0.3224
Pure Error	-	2.305×10^{-5}	-	-	-	0.12	-	-
	S.D. *	3.957×10^{-3}	r^2 *	0.9824	S.D. *	0.50	r^2 *	0.8092
	Mean	0.16	Adj r^2 *	0.9748	Mean	92.57	Adj r^2 *	0.7615
	C.V. % *	2.46	Pred r^2 *	0.9571	C.V. % *	0.54	Pred r^2 *	0.6498
	PRESS *	2.663×10^{-4}	Adeq Precision *	22.711	PRESS *	3.61	Adeq Precision *	8.234

* S.D., standard deviation associated with the experimental error; Mean, dependent mean (average of all the values of the response); C.V. %, coefficient of variance of the model; PRESS, Predicted Residual Sum of Squares for the model; r^2, multiple correlation coefficient; Adj r^2, r^2 adjusted for the number of parameters in the model; Pred r^2, predicted r^2 is a measure of how the model predicts a response value; Adeq Precision, adequate precision is a measure of the range in predicted response relative to its associated error.

Table S2. 2^3 factorial design for the development of spray-dried INH-MOF loaded MP blend and the respective response parameters (gray rows) and additional data for individual PLGA MPs and LC MPs with different MOF concentrations (white rows). For the preparation a hydrophobic and hydrophilic excipient were used: PLGA and leucin. The blends were obtained changing MOF amount, the PLGA/LC ratio, and blending time. The aerodynamic behavior of the blends was assessed through the TSI test by measuring fine particle fraction (%FPF) and emitted dose (%ED). The content homogeneity of the blends corresponding to each run is also reported. Parameters selected by the model for the final formulation and received responses are shown in the blue row.

Run	Factors				Responses		Content Homogeneity p-value
	A	B		C			
	INH-MOF content [% *w/w*]	Blending ratio [% *w/w*]		Blending time [min]	%FPF	%ED	
		PLGA	LC				
**1**	20	20	80	5	48.06	91.29	0.078
**2**	20	80	20	5	25.99	90.91	0.840
**3**	20	20	80	15	46.50	91.90	0.711
**4**	20	80	20	15	26.86	91.85	0.368
**5**	40	20	80	5	47.17	93.19	0.103
**6**	40	80	20	5	28.18	92.00	0.483
**7**	40	20	80	15	54.78	93.30	0.069
**8**	40	80	20	15	28.06	92.97	0.380
**9**	30	50	50	10	52.29	94.01	0.740
**10**	30	50	50	10	45.75	93.35	0.192
**11**	30	50	50	10	49.43	93.50	0.128
**12**	20	100	0	0	11.30	92.53	0.937
**13**	30	100	0	0	23.20	92.52	0.066
**14**	40	100	0	0	11.37	96.34	0.361
**15**	20	0	100	0	53.32	91.99	0.302
**16**	30	0	100	0	48.77	92.10	0.329
**17**	40	0	100	0	52.27	92.42	0.157
**Final formulation**	30	70	70	15	56.65	90.94	0.620

Table S3. Content uniformity data.

Run	Target Content [% *w*/*w*]	Actual Content Mean [% *w*/*w*]	%RSD (*n* = 9)	± 10% of Mean
1	20	24.11	3.73	±2.41
2	20	23.41	3.43	±2.34
3	20	25.04	5.93	±2.50
4	20	23.75	3.74	±2.38
5	40	50.77	7.33	±5.08
6	40	49.66	5.32	±4.97
7	40	46.27	2.70	±4.63
8	40	49.99	1.06	±4.99
9	30	27.09	4.63	±2.71
10	30	31.03	4.43	±3.10
11	30	33.55	4.13	±3.35
